# Evaluation of gold helical microwire structure electrode for long-term rodent nerve stimulation

**DOI:** 10.1088/1741-2552/ade18a

**Published:** 2025-06-18

**Authors:** Danny V Lam, Kevin Yang, Derrick X Liu, Anna Lauricella, Yingyi Gao, Elizabeth S Fielding, Kyle Golobish, Stephan Nieuwoudt, Doug J Weber, Lee E Fisher, Scott F Lempka, Ashley N Dalrymple, Kip A Ludwig, Andrew J Shoffstall

**Affiliations:** 1 Department of Biomedical Engineering, Case Western Reserve University, Cleveland, OH, United States of America; 2 Advanced Platform Technology Center, Louis Stokes Cleveland VA Medical Center, Cleveland, OH, United States of America; 3 Neuronoff Inc., Cleveland, OH, United States of America; 4 Department of Mechanical Engineering and the Neuroscience Institute, Carnegie Mellon University, Pittsburgh, PA, United States of America; 5 Rehab Neural Engineering Labs, Departments of Physical Medicine and Rehabilitation and Bioengineering, University of Pittsburgh, Pittsburgh, PA, United States of America; 6 Departments of Biomedical Engineering and Anesthesiology, Biointerfaces Institute, University of Michigan, Ann Arbor, MI, United States of America; 7 Departments of Biomedical Engineering and Physical Medicine and Rehabilitation, University of Utah, Salt Lake City, UT, United States of America; 8 Wisconsin Institute for Translational Neuroengineering (WITNe), Department of Neurological Surgery, Department of Surgery, University of Wisconsin—Madison, Madison, WI, United States of America

**Keywords:** percutaneous leads, peripheral nerve stimulation, neuromodulation, tissue encapsulation, electromyography

## Abstract

*Objective.* The development of electrodes for chronic peripheral nerve stimulation faces several challenges, including complex compositions, intricate manufacturing processes, and high costs associated with the availability and fabrication of suitable materials. These limitations hinder the accessibility and feasibility of producing effective devices for chronic preclinical studies. This study evaluated the feasibility of a simple-to-manufacture gold helical microwire structure electrode (Au-HMSE) for peripheral nerve stimulation, electromyography (EMG) recording, and preliminary tissue response on the rat sciatic nerve. *Approach.* Manufactured electrodes were used for up to 8 weeks in rats for nerve stimulation and EMG recordings, with electrode-tissue impedances and motor thresholds measured to assess *in vivo* stability and feasibility. Evoked motor responses were measured via gastrocnemius muscle activity and ankle torque. Terminal histology was performed at 12 weeks to assess chronic tissue response to the implanted electrodes. *Main results.* Implanted electrodes with impedances <10 kΩ effectively evoked motor responses in monopolar and bipolar configurations and successfully recorded EMG activity. Gastrocnemius activation overlapped with off-target motor responses, likely due to the electrode’s size relative to rat nerve anatomy and the absence of anchoring, which may have allowed migration. High impedance failure appeared related to interconnects between electrodes and tunneled leads and at solder joints in the stimulating and recording setup. Histology showed typical fibrotic encapsulation, with the helical design promoting tissue in-growth around the microwires, creating a high surface area electrode-tissue interface. *Significance.* This study evaluated the early feasibility of Au-HMSE for chronically implanted rodent nerve stimulation and EMG recordings. While gold electrodes are non-standard for chronic stimulation, the construction of these devices may be appropriate for the evaluation of chronic peripheral nerve stimulation in the preclinical setting due to their simple composition, manufacturing, and availability of gold microwire as a raw material. The findings provide valuable insights for developing future implantable leads used for peripheral nerve stimulation.

## 1. Introduction

The development of neural interfaces for neuromodulation of peripheral nerves has garnered significant attention in recent years due to its potential in treating various neurological disorders. For example, vagus nerve stimulation (VNS) has been explored for its therapeutic effects in epilepsy, depression, obesity, and stroke rehabilitation (Johnson and Wilson 2018, Goggins *et al* 2022). Similarly, tibial nerve stimulation has been employed in managing an overactive bladder (Bhide *et al* 2020, McPhail *et al* 2023). Additionally, the stimulation of multiple peripheral nerves has been investigated as a means to alleviate chronic pain at various areas of the body, including suprascapular and axillary shoulder, femoral and genicular knee, and occipital migraine (Helm *et al* 2021, Kaye *et al* 2021, Busch *et al* 2022). While the diverse applications of peripheral neuromodulation demonstrate its potential in treating several neurological disorders, the effectiveness of neuromodulation therapy heavily depends on the design and reliability of the neural interface used. Various electrode and lead designs have been developed to restore function and reduce symptoms of numerous neurological disorders and meet specific clinical and anatomical requirements (Grill *et al* 2009, De Raedt *et al* 2015, Christie *et al* 2017, Deer *et al* 2020, Won *et al* 2020). For example, trifilar coils insulated with polymer tubing are frequently utilized as leads due to their flexibility and durability, enabling them to withstand numerous loading and unloading cycles before experiencing mechanical failure (Abler *et al* 1992, Nisam and Reddy 2015, Toossi *et al* 2017). Commonly used neural interfaces include designs such as ring leads, initially developed for deep brain stimulation, and cuff and spiral electrodes wrapped around the nerve to achieve a stable and close interface (Paggi *et al* 2021, Frey *et al* 2022).

Despite design improvements, peripheral neural interfaces often encounter problems such as lead breakage and migration (Eldabe *et al* 2016, Hoffmann *et al* 2023). Lead breakage may occur when the device is exposed to repeated stress or bending, which can compromise the integrity of the electrical connection and the ability to deliver an effective charge to stimulate nerves (Hoffmann *et al* 2023). On the other hand, lead migration refers to the physical displacement of the electrode from the target nerve, leading to reduced effectiveness or unintended stimulation of adjacent tissues (Sharan *et al* 2015, Eldabe *et al* 2016).

Previous studies have successfully incorporated helical structures in percutaneous lead designs, demonstrating potential benefits such as improved tissue anchoring, reduced lead migration, lower infection rates, and enhanced overall device functionality (Daly *et al* 2001, Ilfeld *et al* 2017a). Helical-based electrodes, constructed from tightly wound microwires, offer a promising solution to issues related to lead breakage and migration. Our group has recently demonstrated the benefits of this architecture when removing the devices, namely sustained low pull-out forces (Howe *et al* 2024). Further, these electrodes are highly flexible, capable of withstanding significant strain deformation before failing, and are resistant to electrode ‘pistoning’, a common cause of migration (Ilfeld *et al* 2017a, Gabriel *et al* 2019). When used in an augmented transcutaneous configuration, these leads have the potential to be formed into a two-component system, comprising both lead and insulated electrode contact in one device, without any interconnects (Verma *et al* 2021).

This study evaluated the use of helical-based electrode comprised of gold microwires (Au-HMSE) as a neural interface and their long-term performance in a small animal rodent model through stimulation of the rat sciatic nerve. Although it was presumed that Au-HMSE would serve as an effective neural interface for peripheral neuromodulation, this electrode design had yet to be directly evaluated in small animal models. The rat’s sciatic nerve is a common target in pre-clinical peripheral neuromodulation studies because it is one of the largest peripheral nerves in rats. Assessing the resulting motor response is a well-established and relatively straightforward procedure (Lam *et al* 2023). The primary outcomes of this study include the measurement of electrode-tissue impedance, evoked motor responses from contracting hind limb muscles and ankle flexion, and post-mortem histology of the implant site.

## 2. Materials and methods

### 2.1. Electrode manufacturing and assembly

**Figure 1.**
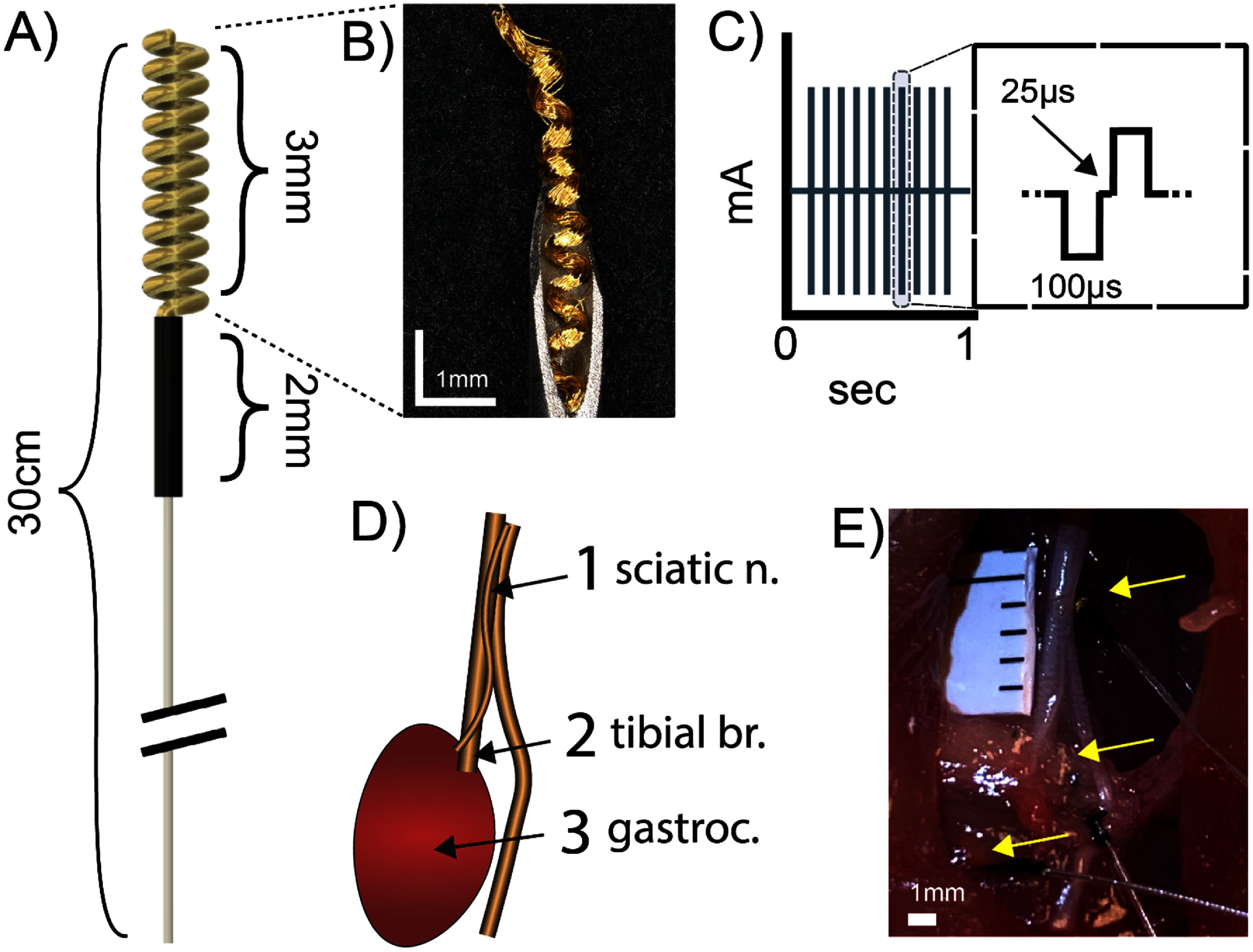
Experimental setup for rat sciatic nerve stimulation and electromyography recording. (A) A schematic representation of the Au-HMSE. The total length of the percutaneous lead was 30 cm, providing sufficient slack during the tunneling procedure. (B) Au-HMSEs were imaged before implantation to detect potential defects. (C) A cathodic-leading biphasic stimulation waveform was used for electrical stimulation of the sciatic nerve. (D) A diagram illustrating the locations of implanted Au-HMSEs under the sciatic nerve (rostral electrode), at the sciatic nerve trifurcation (caudal electrode), and within the gastrocnemius muscle. (E) Representative image, mirroring panel D, showing the position of surgically implanted leads in a rat’s hind limb.

Au-HMSEs were manufactured by Neuronoff Inc. (OH, USA) in a previously published method (Howe *et al* 2024). The helical microwire structure was manufactured from 25 *μ*m diameter 99.99% pure gold wire (Heraeus, Hanau, GER) and mechanically coupled to a stainless-steel lead wire (Cooner Wire, CA, USA) (figures 1(A) and (B)). Polyolefin heat shrink tubing (McMaster-Carr, OH, USA) was used to secure the transition site of the helical microwire structure and the lead wire for a total length of 30 cm. The uninsulated section of the electrode assembly had dimensions of 3 mm in length and 0.6 mm in diameter (outer diameter of helix). The total exposed surface area was estimated at 0.222 cm^2^ based on a cylindrical shell model. The assembled Au-HMSE was loaded into a standard 19-gauge needle (EXELint, CA, USA) and a 21-gauge blunt needle pin (McMaster-Carr, OH, USA) (figure 1(B)). Assembled electrodes were sterilized through standard ethylene oxide protocols before implantation (Cleveland Clinic, OH, USA). Preliminary cyclic voltammetry data demonstrated a large cathodic charge storage capacity (883 ± 49.4 mC cm^−2^) for samples not used for animal study (*n* = 3; figure S3).

### 2.2. Animal use and surgical procedures

All surgical and experimental procedures were performed with the approval and oversight of the Case Western Reserve University Institutional Animal Care and Use Committee, and in accordance with the Guide for the Care and Use of Laboratory Animals, as well as all applicable federal, state, and local animal welfare regulations and the ARRIVE guidelines (National Research Council 2011).

A rat model was implemented to evaluate the long-term performance and stability of the implanted microwires as a neural interface (figure 1(D)) (Atkinson *et al* 2022). Sprague-Dawley rats (*n* = 4) received bilateral implantation of Au-HMSEs for sciatic nerve stimulation and intramuscular electromyography (EMG) recording to assess electrode feasibility for chronic *in vivo* applications. The rats were anesthetized with vaporized isoflurane at 4% and tapered to 1.5%–2% to maintain a surgical plane of anesthesia. Prior to surgery, lidocaine (up to 10 mg kg^−1^) was subcutaneously administered at incision sites of the subject’s hind limbs, scalp, and along the back, where leads would be tunneled. A 2.5 cm midline incision of the scalp was performed to expose the skull. A superficial (partial) burr hole craniotomy sufficient to establish an anchor in the skull was performed using a dental drill at 15 000 RPM with saline washes in between 2 and 5 s intervals. A stainless-steel bone screw with a diameter of 1.59 mm (Stoelting Co., IL, USA) was fixed into the skull to serve as an anchor for securing the connector containing lead wires to each electrode. The head cap was secured to the skull with cold cure dental cement, which was applied to the implanted stainless-steel screw. Skin incisions were closed with monofilament sutures. Rats received meloxicam (2 mg kg^−1^) and buprenorphine (0.03 mg kg^−1^) for post-operative pain management.

A lateral skin incision was made adjacent to the femur on the hind limb for electrode implantation. Afterward, the vastus lateralis and bicep femoris muscles were separated to expose the sciatic nerve. An Au-HMSE was implanted via a syringe and needle using a smaller gauge needle as a plunger under the sciatic trifurcation and was treated as the caudal lead (towards calf muscles) (figures 1(D) and (E)). A second Au-HMSE was implanted ∼1–2 cm towards the rostral direction (towards the spine) under the main sciatic nerve branch, oriented perpendicular to the direction of the nerve. A third Au-HMSE was implanted within the belly of the gastrocnemius lateralis muscle (figure 1(E)). Surgical procedures and electrode implantations were repeated on the contralateral hind limb. Stainless-steel wires (Cooner Wire, CA, USA) served as reference and ground wires for electrode-tissue impedance measurements. Reference wires were placed in nearby adipose tissue of respective hind limbs. The ground wire was placed in the adipose tissue of the back, ∼5 cm distal to reference wires. Leads were subcutaneously tunneled from the hind limb incision site towards the scalp along the back, soldered onto a 12-pin connector (Omnetics Connector Corporation, MN, USA) of the head cap, and insulated with silicone adhesive (Kwik-Cast, World Precision Instruments, Inc., FL, USA).

For electrode-tissue impedance measurements, the connector pins corresponding to the reference and ground wires were connected to respective inputs of a passive four-channel adapter (SB4, Tucker–Davis Technologies, FL, USA) attached to a data acquisition system (Lab Rat, Tucker–Davis Technologies, FL, USA). Electrode-tissue impedance measurements were collected with the accompanying recording software (SynapseLite, Tucker–Davis Technologies, FL, USA) using an input sinusoidal wave at ∼1 kHz frequency and 9 nA root-mean-square amplitude (Lab Rat, Tucker–Davis Technologies, FL, USA). A threshold impedance value of >10 kΩ was selected to identify non-viable electrodes for electrical nerve stimulation due to mechanical or biological issues, such as faulty interconnects or fibrotic encapsulation. This cutoff value was selected based on the electrode’s ability to evoke muscle contraction during nerve stimulation, as reported in previous chronic studies (Payne *et al* 2020, Mughrabi *et al* 2021).

### 2.3. Sciatic nerve stimulation

Following electrode implantation, animals underwent testing sessions at weeks 1, 2, 3, 4, and 8 post-surgery. During each session, the animal was anesthetized with isoflurane, and measurements of torque, EMG, and electrode-tissue impedance were performed. No testing was conducted during weeks 5–7 and 9–12 due to personnel availability and institutional closures during the SARS-CoV-2 pandemic. An overview of the experimental timeline is provided in table 1.

**Table 1.** Experimental timeline.

Week	0	1	2	3	4	8	12
Bilateral Electrode Implantation Surgery (*N* = 4)	X						
Stimulation and Recording Sessions (Evoked Ankle Torque, EMG)		X	X	X	X	X	
Endpoint Histology							X

Both bipolar and monopolar stimulation configurations were investigated one week after electrode implantation. The bipolar stimulation configuration used the rostral and caudal electrodes as the anode and cathode, respectively (figures 1(D) and 4). For monopolar stimulation, the rostral or caudal electrode was selected as the cathode, while the ground electrode was set as the anode. Charge-balanced symmetric biphasic pulses (cathodic-leading) were delivered to the sciatic nerve at stimulation amplitudes corresponding to the subject’s motor threshold using an AC/DC source (Keithley Instruments, OH, USA). Stimulation was delivered at a frequency of 10 Hz, 100 *μ*s pulse width, and 25 *μ*s inter-phase delay for a 1 s stimulation train (figure 1(C)). Dose-response curves were established by gradually raising the stimulation amplitudes from the motor threshold at increments of 0.1 mA for up to six iterations or reaching a maximum stimulation amplitude of 2.5 mA.

### 2.4. Data collection and signal processing

#### 2.4.1. Stimulation-evoked ankle torque

A hind limb stabilization apparatus was previously developed to secure the rat’s hip, knee, and ankle into position for isometric muscle contraction (Lam *et al* 2023). Subjects were positioned in the apparatus under anesthesia. The subject’s knee and hip were positioned at 90° and secured into the knee-locking assembly with the provided shafts and clamps equipped with tightening screws. The subject’s foot was positioned at 90° and secured onto the foot pedal equipped with a torque transducer. Stimulation-evoked ankle torque was digitized at 10 Hz via a DE19-P connector (JR3 Inc., CA, USA) to a 16-bit analog-to-digital converter (NI-DAQ Model 9222, National Instruments Inc., TX, USA), followed by baseline detrending. Absolute peak torque was recorded.

#### 2.4.2. Electrophysiology recording

Electrophysiology signals were digitized at ∼25 kHz with a passive four-channel adapter (SB4, Tucker–Davis Technologies, FL, USA) attached to a data acquisition system (Lab Rat, Tucker–Davis Technologies, FL, USA).

A custom Python (Version 3.7) script was written for signal processing following similar reported filtering steps (Blanz *et al* 2023, ludwig-lab/pyeCAP 2023). In brief, the difference between the raw and median filtered signal (kernel size: 201) was passed through a Gaussian filter (*σ* = 0.001). A finite impulse response filter at 60, 120, 180, and 240 Hz was used to remove powerline noise with 1.0 Hz bandwidth. Stimulation artifacts for a given stimulation train were detected via peak thresholds for all recording channels. EMG signals were quantified as the area-under-the-curve within an 8 ms time window following the stimulation artifact.

In demonstrating that evoked activity was physiological, EMG recordings were collected from a rat (not elsewhere included in the study) 20 min after euthanasia (figure S1). Minimal muscle activity was detectable post-euthanasia, possibly evoked from residual excitable fibers in the gastrocnemius muscle (figure S1(C)). Previous studies have shown that central nervous system neurons stop functioning within 5–15 min after euthanasia if they are not promptly excised and placed in an oxygen- and nutrient-rich medium (Heymans 1950, Deshmukh *et al* 2024). According to Elmas *et al*, the muscle activity in response to electrical nerve stimulation will decrease over time after euthanasia and become unresponsive after 40 min (2001).

### 2.5. Tissue processing and histology

Twelve weeks after implantation, rats were anesthetized via intraperitoneal injections of ketamine (160 mg kg^−1^) and xylazine (20 mg kg^−1^), followed by transcardial perfusion of phosphate-buffered saline solution and 10% neutral buffered formalin. Nerve, muscle, and subcutaneous skin tissues containing electrodes were extracted and post-fixed in 10% neutral buffered formalin for 24 h. Tissues were stored in a 70% ethanol solution for at least 24 h before paraffin embedding. Tissues were sectioned at 10 *μ*m and stained with Masson’s trichrome, hematoxylin, and eosin (H&E) solutions to assess fibrosis and immune cell infiltration. Slides containing tissues were imaged with an automated digital slide scanner (Axio Scan.Z1, ZEISS, GER) with a 20x/0.8 plan-apochromatic objective (ZEISS, GER). Brightfield images were captured at 1.5% light source intensity and a 2 ms exposure period.

### 2.6. Statistics

A custom Python script (Version 3.7) was used to perform all statistical analyses for this study. Linear correlation was performed between motor threshold values and electrode-tissue impedances. Student’s t-test was performed to evaluate differences in the evoked muscle activity from recorded EMG for contracting muscles versus contralateral muscles and pre- versus post-euthanasia measurements. Data distributions were observed to be roughly symmetric; no gross violations of normality or homogeneity of variance were evident that would necessitate non-parametric alternatives. Statistical significance was represented as: * = *p* < 0.05, ** = *p* < 0.01, *** = *p* < 0.005. All data were presented as mean ± standard error of the mean (SEM). No formal power analysis was conducted due to the nature of this feasibility study and the practical constraints on animal use.

## 3. Results

Key metrics used to evaluate implanted Au-HMSEs for neuromodulation included stimulation motor thresholds, electrode-tissue impedances, and quantification of the evoked motor responses (intramuscular EMG from the gastrocnemius muscle and ankle torque). Additionally, EMG was recorded using the Au-HMSEs to demonstrate additional utility for future applications. Stimulation motor thresholds were defined as the minimum stimulation amplitude in which evoked ankle flexion was observed using an ankle torque apparatus. Both monopolar and bipolar stimulation configurations were explored to assess their effectiveness in eliciting a motor response. This comprehensive evaluation process helps to confirm the key features of the Au-HMSEs and their potential applications for expanding neuromodulation techniques, especially for clinical interventions and management of refractory chronic pain.

### 3.1. Implanted electrodes demonstrate robust ‘viability’ over 8 weeks

While there are several other common electrochemical tests to evaluate electrode performance (e.g. voltage transients), here we chose to assess electrode impedance (at 1 kHz) as a rapid measurement of electrode ‘viability’ (Cogan 2008, Wilks *et al* 2017). Specifically, a threshold of >10 kΩ, or ∼5x higher than our baseline impedance, was selected to detect lead or interconnect breakages or other major device failures.

**Figure 2.**
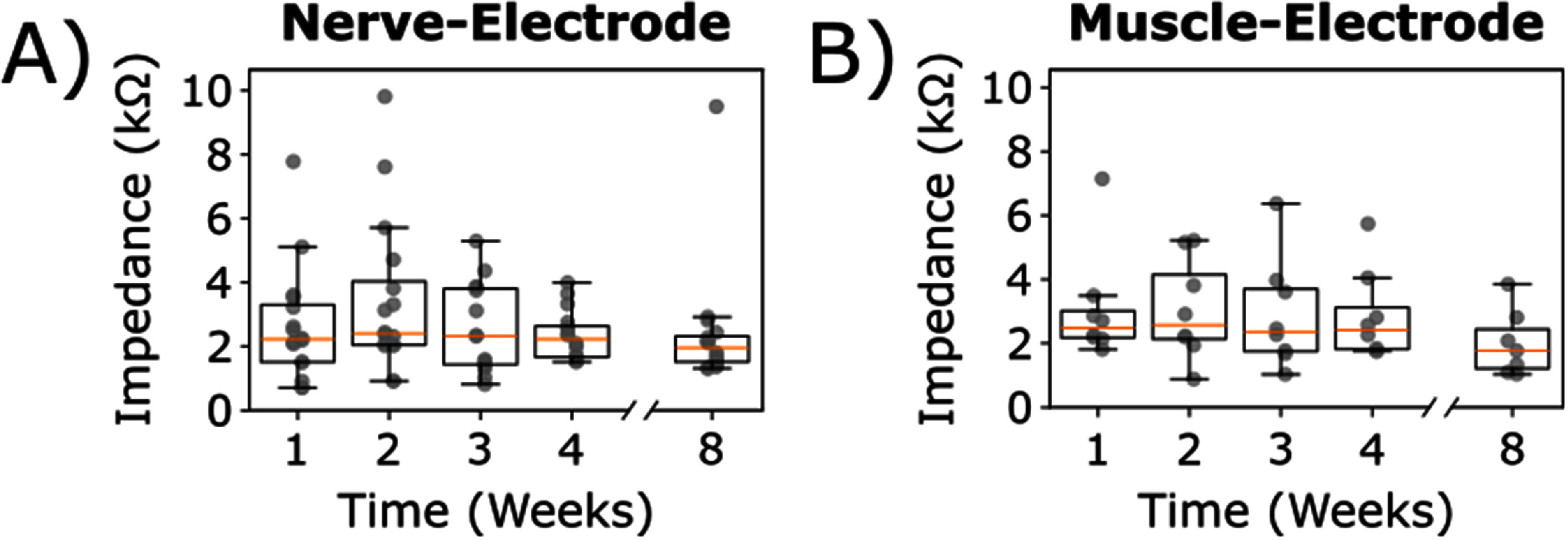
The implanted Au-HMSEs demonstrated robust stability over time, as evidenced by their *in vivo* electrode-tissue impedance. (A) The electrode-tissue impedances were recorded for all implanted Au-HMSEs placed under the sciatic nerve, under the sciatic trifurcation, and within the gastrocnemius muscle for up to 8 weeks. There were no substantial changes observed in the electrode-tissue impedances for each respective electrode. Orange lines, black boxes, and whiskers represent medians, interquartile ranges, and maximum and minimum values, respectively.

In this study, all electrode-tissue impedances remained <10 kΩ for up to 2 weeks and increased afterward (figure 2). By week 8, 92% (22/24) of the implanted electrodes were determined to be viable based on this criterion. Among those implanted under the sciatic nerve (rostral electrode) and its trifurcation (caudal electrode), 93% (15/16) were viable. The average electrode-tissue impedance for electrodes implanted on nerve was 2.75 ± 0.31 kΩ at baseline, while those in the gastrocnemius muscle measured at 2.68 ± 0.16 kΩ. Throughout this study, two implanted electrodes were deemed ‘non-viable’ for neural interfacing with electrode-tissue impedances >10 kΩ. These non-viable electrodes included one placed under the sciatic trifurcation, which became non-viable at week 3, and the other placed within the gastrocnemius muscle, which became non-viable at week 8.

### 3.2. Stimulation-evoked responses measured by intramuscular EMG recordings

**Figure 3.**
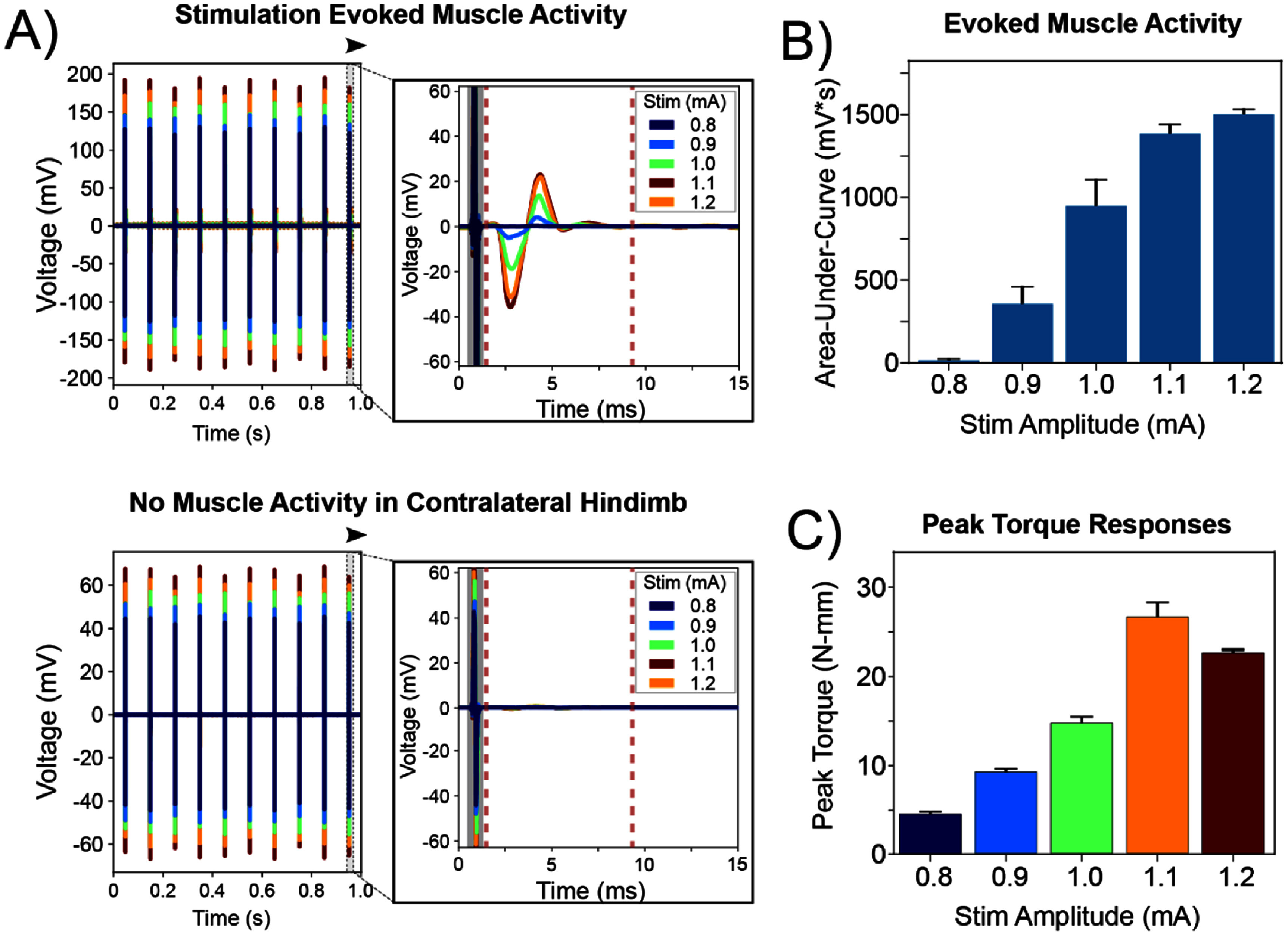
Representative EMG activity in response to sciatic nerve stimulation (*n* = 1). Evoked muscle activity was demonstrated in EMG recordings from electrodes implanted in the gastrocnemius muscles of both hind limbs. (A) The recorded stimulation trains at increasing stimulation amplitudes were plotted from the stimulated hind limb (top) and contralateral hind limb (bottom). EMG recordings showed distinct waveforms following the stimulation (Stim) artifact (grayed) within the contracting gastrocnemius muscle. In contrast, no distinct muscle activity was observed in the contralateral hind limb. (B) The signals were quantified as area-under-the-curve within an 8 ms time window (dashed red vertical lines). Evoked muscle activity increased significantly for stimulation amplitudes above the threshold amplitude (0.8 mA). (C) Ankle torque significantly increased for stimulation amplitudes above the threshold (0.8 mA). All data are represented as mean ± SEM.

EMG recordings were used to demonstrate evoked muscle activation in response to electrical stimulation of the sciatic nerve, as shown by a representative subject in week 1 (figure 3(A) and (B)). Sciatic nerve stimulation was achieved using charge-balanced pulses with a bipolar configuration (cathode on caudal lead, anode on rostral lead). Within EMG recordings, evoked muscle activity was observed following the stimulation artifact in the stimulated hind limb (figure 3(A), top). No activity was observed in the unstimulated hind limb, which helped to validate that the activity in the stimulated hind limb were physiological and not influenced by noise or artifacts (figure 3(A), bottom).

The evoked muscle activity in response to electrical stimulation was measured within an 8 ms time window following the stimulation artifact. The results showed increasing amplitudes of evoked muscle activity with increasing stimulation amplitudes (0.8–1.2 mA) (figure 3(B)).

Ankle torque measurements were used to complement EMG recordings and determine motor thresholds, which were found to be 0.8 mA for this representative subject (figure 3(C)) (Lam *et al* 2023). Furthermore, concurrent measurements of ankle torque, conducted alongside EMG recordings in the stimulated hindlimb, showed increased torque output (N-mm) in relation to increasing stimulation amplitudes (figure 3(C)).

### 3.3. Electrical nerve stimulation can chronically evoke motor responses up to 8 weeks

**Figure 4.**
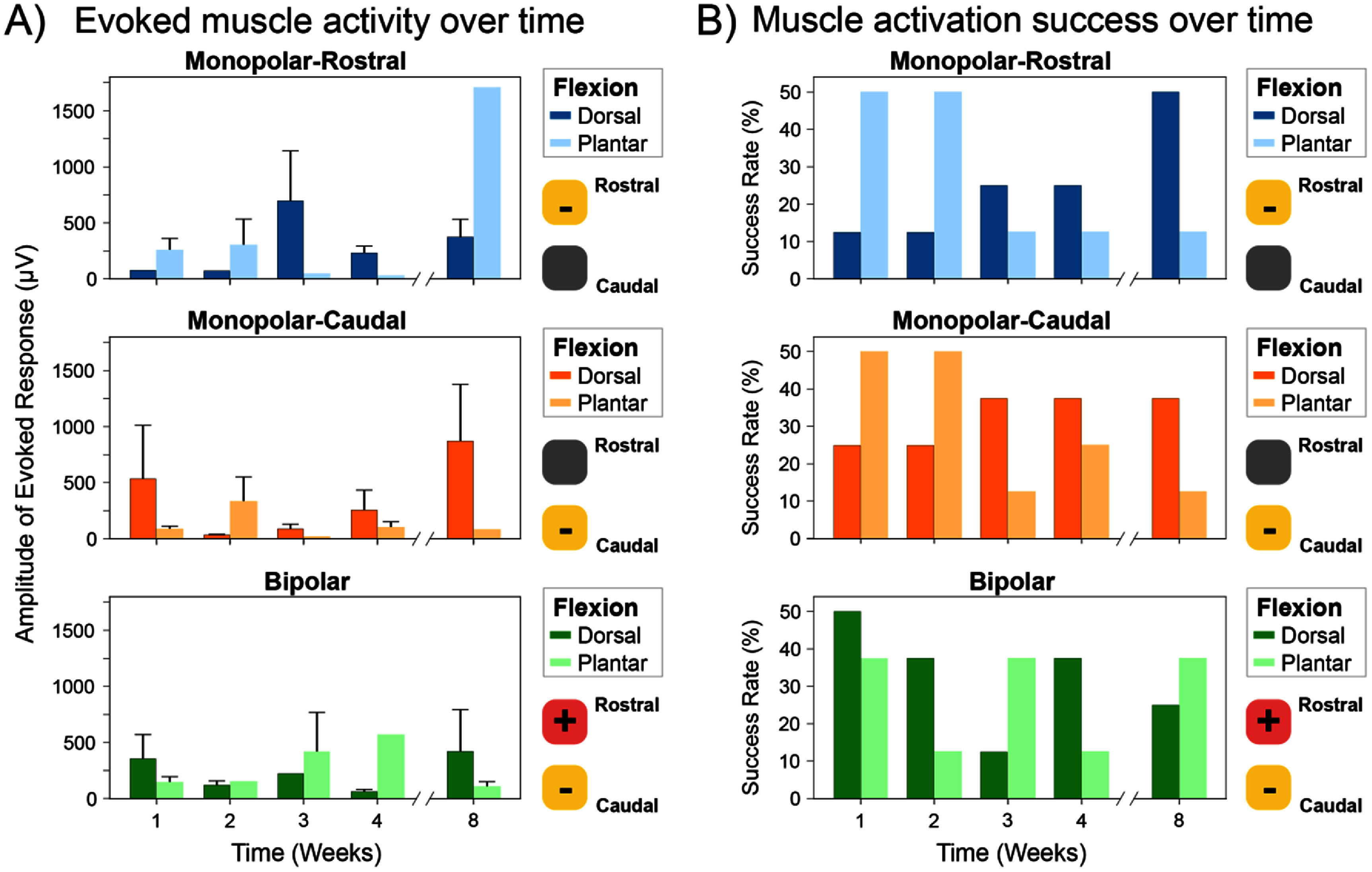
Chronic sciatic nerve stimulation results in variability in intensity and specificity for evoked motor activation, as seen by inconsistent evoked muscle activity of the gastrocnemius muscle and different ankle flexions. (A) Monopolar (cathode used as either rostral or caudal electrode) and bipolar stimulation configurations were evaluated for feasibility and to identify if any preferences for either stimulation configuration were observed in evoking gastrocnemius muscle activity. Furthermore, the ankle flexion (dorsal or plantar) was measured with confirmed muscle activity in EMG recordings. The evoked muscle activity was quantified as the area-under-the-curve within an 8 ms time window following the stimulation artifact. In addition, there were no discernable trends in the signal intensity for EMG recordings with the evoked ankle flexion. (B) Confirmation of evoked muscle activity in the gastrocnemius muscle, shown as a success rate percentage when accompanied by an evoked ankle flexion, was measured to identify any observable patterns associated with the evoked ankle flexion. However, the results showed no consistent preferences in ankle flexion associated with confirmed muscle activity in the gastrocnemius muscle. All data were represented as mean ± SEM.

Intramuscular EMG recordings are a commonly used method to monitor the efficacy of sciatic nerve stimulation. In demonstrating the *in vivo* performance of chronically implanted AU-HMSEs, EMG was recorded with respect to various stimulation configurations, including monopolar (cathode used as either the rostral or caudal electrode) or bipolar (figure 4). Initially, EMG recordings were used for continuous monitoring to detect signal decay, which could result from a decline in electrode performance, such as reduction in nerve stimulation efficacy or signal decay in EMG recording. However, the signal quality in EMG recordings varied over the 8 weeks without consistent trends (figure 4).

**Figure 5.**
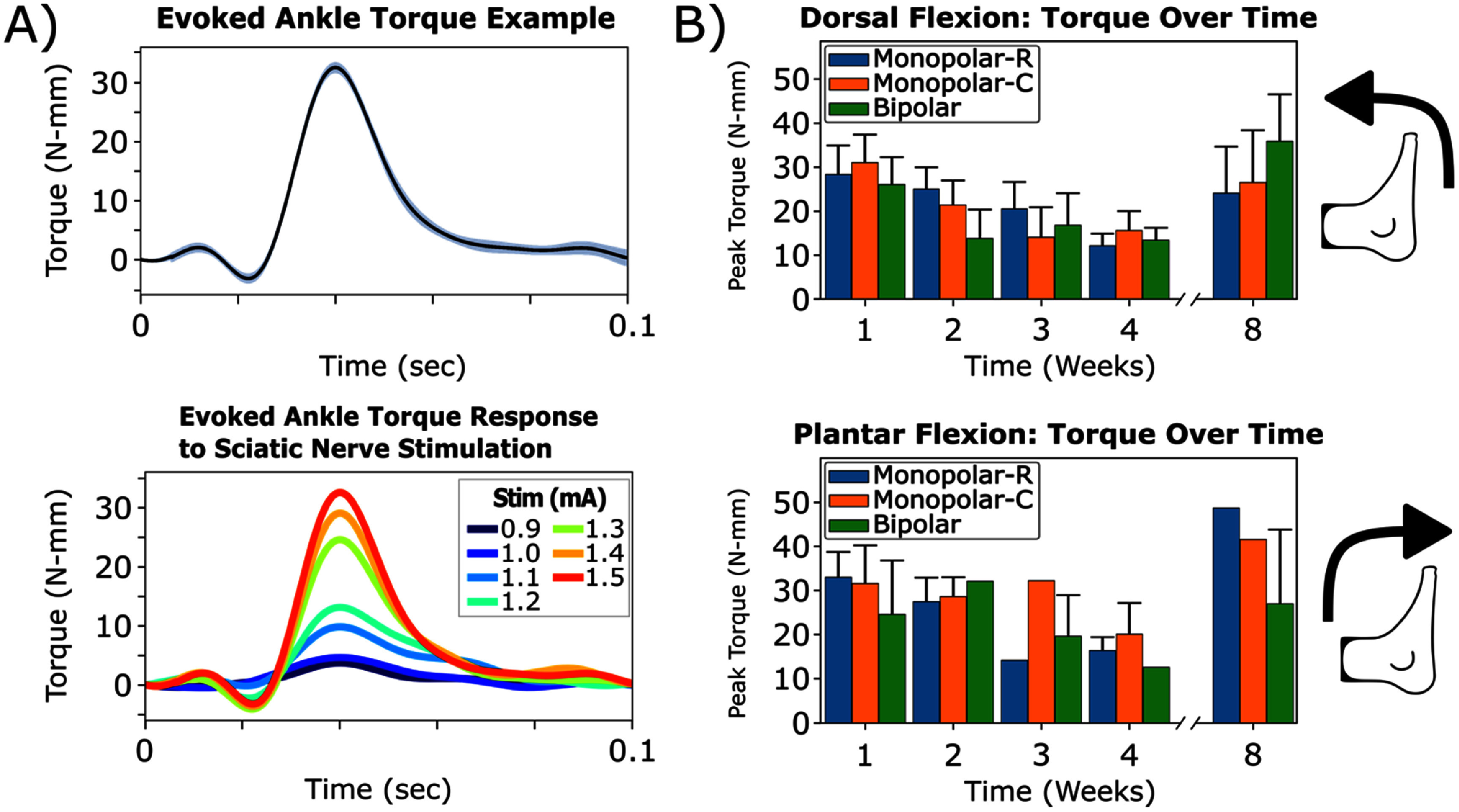
Electrical stimulation of the sciatic nerve evoked ankle flexion towards the dorsal or plantar direction, with no apparent bias towards a specific stimulation configuration. (A) Example torque measurements from plantar flexion are shown from a representative subject (*n* = 1) at a stimulation amplitude of 1.5 mA (top) and between 0.9 and 1.5 mA (bottom). (B) Monopolar (rostral and caudal represented as ‘-R’ and ‘-C,’ respectively) and bipolar stimulation configurations were evaluated for feasibility and whether any preferences occurred for evoking a specific direction of ankle flexion for any stimulation configuration. All data were represented as mean ± SEM.

To address these discrepancies and gain additional insights into the efficacy of the Au-HMSEs for electrical nerve stimulation, we incorporated torque measurements of the ankle as a complementary metric to evaluate the effects of stimulation. In addition to the direction of the ankle flexion (dorsal or plantar), peak torque was recorded for the evoked ankle flexion, as observed in a representative subject (figures 5(A) and S2). For this representative subject, the increased peak torque was observed to correspond to increasing stimulation intensity (figure 5(A), bottom). The direction for ankle movement and the peak torque at respective motor thresholds were recorded up to week 8 for each stimulation configuration (figure 5(B)). Of 144 potential observations, 121 measurements yielded an ankle response (dorsal flexion: 69/121; plantar flexion: 52/121). The subset of subjects in which stimulation at this maximum amplitude did not drive a response mostly likely had electrode migration or positional effects from the electrode shifting with respect to the nerve. Similar to inconsistencies observed in the EMG recordings (figure 4), peak torque demonstrated no observable trends regarding the direction of ankle flexion. In some cases, ankle torque magnitudes decreased with increases in amplitude, further validating the presence of antagonistic muscle activation.

### 3.4. Motor thresholds remained consistent for most implanted electrodes

**Figure 6.**
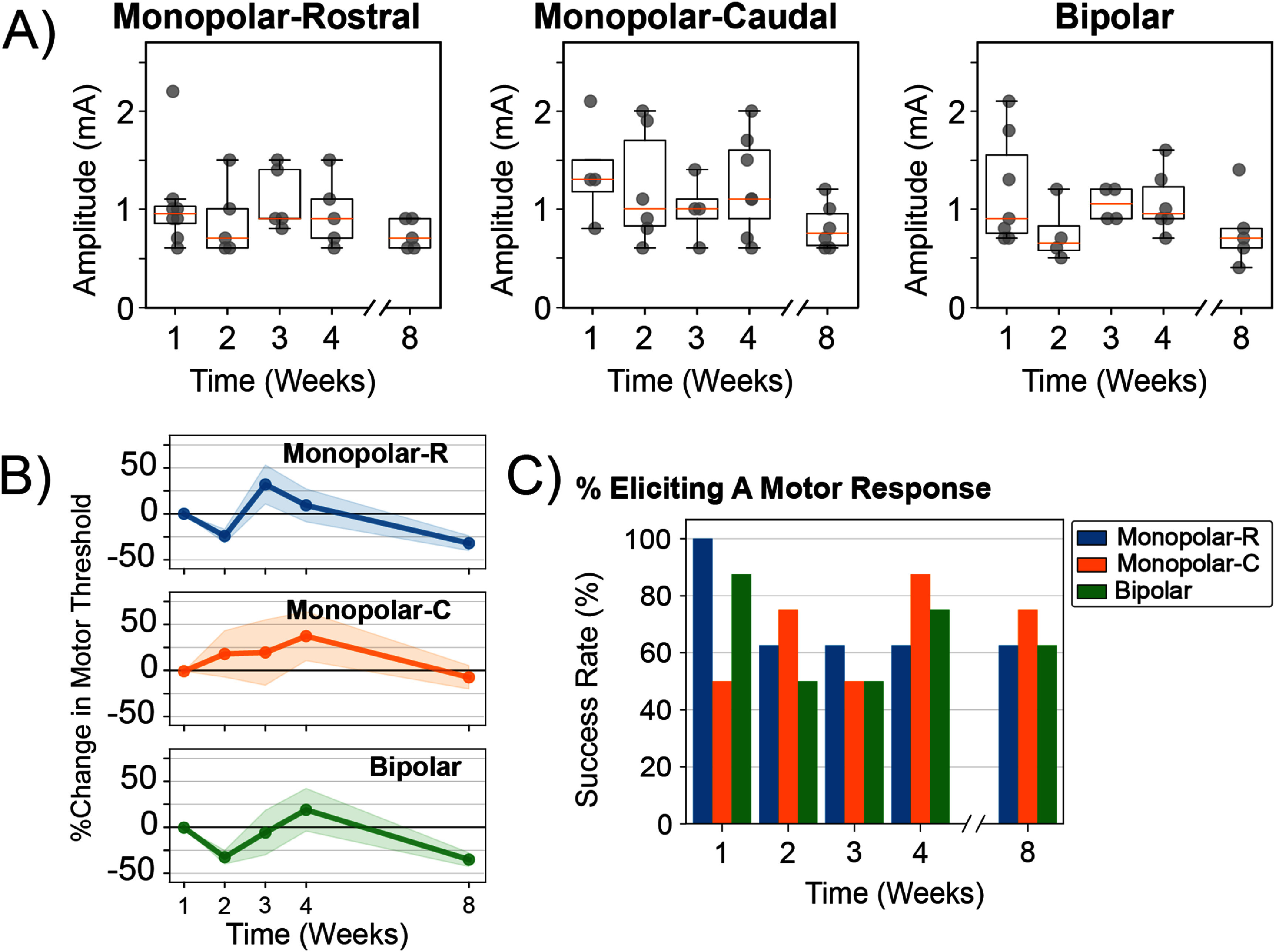
Monitoring changes in evoked motor thresholds over time using electrical nerve stimulation from implanted Au-HMSEs. Both monopolar and bipolar stimulation configurations were evaluated to demonstrate the feasibility and whether any preferences emerged for either configuration in evoking a motor response. (A) Both monopolar (at rostral and caudal electrodes) and bipolar stimulation configurations were investigated for up to 8 weeks. Stimulation amplitudes used to evoke motor responses remained stable for each respective stimulation configuration. Orange lines, black boxes, and whiskers represent medians, interquartile ranges, and maximum and minimum values, respectively. (B) Additionally, minimal deviations from the baseline motor thresholds (at Week 1) were observed, with motor thresholds trending below their initial levels by week 8. (C) Towards the end of the study, there was an observable preference for evoking a motor response using a monopolar-caudal stimulation configuration.

The chronic reliability of implanted Au-HMSEs for electrical nerve stimulation was demonstrated by measuring the motor threshold, which is the minimum stimulation amplitude required to evoke a torque at the ankle (figures 6(A) and (B)). Although the ability to evoke a motor response was sustained throughout the study in the majority of subjects (68% for either monopolar stimulation configuration), the success rates in evoking a motor response varied or declined throughout the 8 weeks (figure 6(C)). Here, the average stimulation amplitude necessary to evoke a motor response varied only minimally based on the configuration (monopolar-rostral: 0.93 ± 0.07 mA; monopolar-caudal: 1.11 ± 0.09 mA; bipolar: 0.96 ± 0.07 mA) (figure 6(A)). However, the change in motor thresholds for respective subjects was observed to fluctuate over time with a decreasing trend for monopolar-rostral and bipolar stimulation configurations (monopolar-rostral: −7.16 ± 11.2%; monopolar-caudal: 10.5 ± 7.82%; bipolar: −15.8 ± 10.2%) (figure 6(B)). By Week 8, the motor thresholds for all stimulation configurations were below their respective baseline measurements (monopolar-rostral: −31.9 ± 8.04%; monopolar-caudal: −6.57 ± 12.7%; bipolar: −34.7 ± 7.53%).

Measurements where a motor response was not elicited for a given week were excluded from the analysis but were accounted for in calculating the success rate for each stimulation configuration over time (figure 6(C)). At week 1, all electrodes (8/8) in the monopolar-rostral configuration successfully evoked a motor response, which decreased to 63% (5/8) by Week 8. Similarly, the bipolar configuration demonstrated best performance at Week 1, with 88% (7/8) of the electrodes successfully evoking a motor response, which declined to 63% (5/8) by week 8. In contrast, the monopolar-caudal configuration exhibited a different trend where 50% (4/8) of the electrodes evoked a motor response at week 1, which increased to 75% (5/8) by week 8. Additionally, the monopolar-caudal stimulation configuration had performed best at week 4, in which 88% (7/8) of the electrodes successfully evoked a motor response. Although each stimulation configuration had varying degrees of success, motor activation was achieved at >62% with either method by week 8.

To investigate whether this decline in motor response was related to the electrode-tissue impedance, a linear regression was performed (using the monopolar configuration data). The electrode-tissue impedance alone does not appear to accurately predict the ability to evoke a motor response as a weak correlation was measured (*r* = 0.17; *p* < 0.08). This implies that our inability to initiate motor activation for those electrodes during the testing sessions was likely caused by other factors, such as electrode migration.

### 3.5. Histological overview of implanted Au-HMSEs

**Figure 7.**
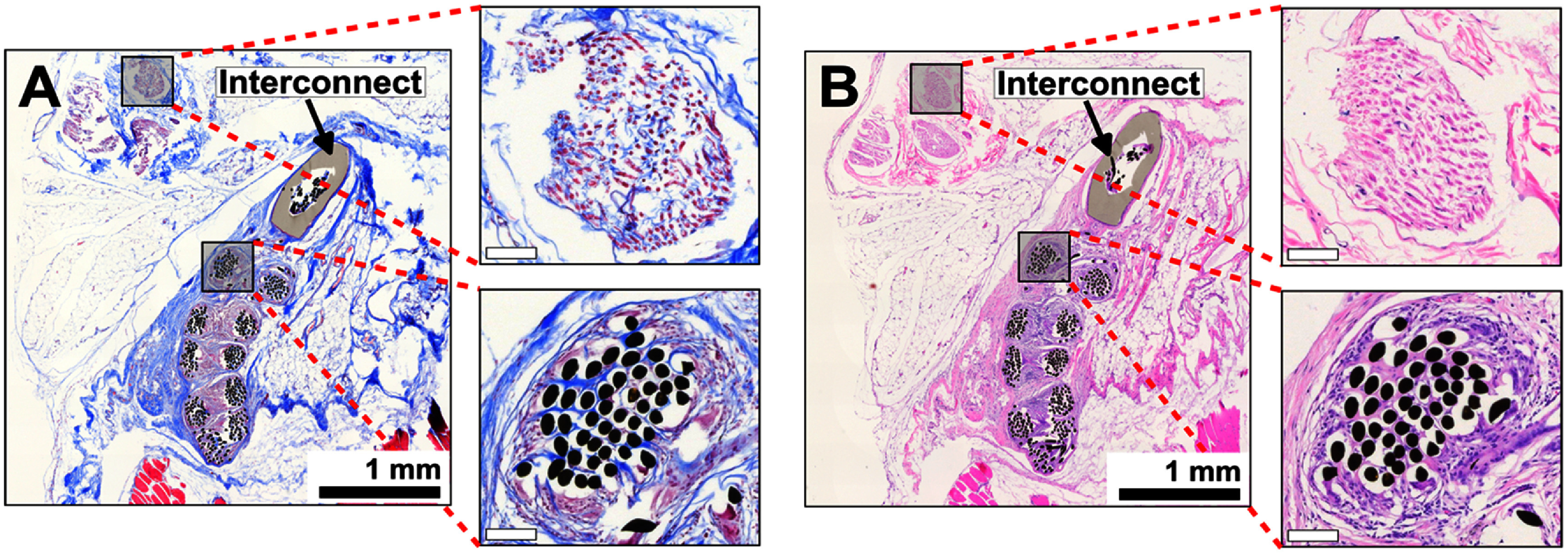
Histology at week 12 reveals the presence of fibrotic tissue encapsulation and infiltration of collagen and immune cells throughout the Au-HMSEs. Representative images of the sciatic nerve fascicle (top zoom panel) and the chronic foreign body response around and within the bundle of Au-HMSEs (bottom zoom panel) are demonstrated through (A) Masson’s trichrome and (B) H&E staining; scale bar = 1 mm. A black arrow points to the interconnect used to connect the Au-HMSE and the lead stainless-steel wire.

After 8 weeks of sciatic nerve stimulation, subjects remained housed for an additional 4 weeks. At week 12, both the sciatic nerve and the surrounding tissue containing implanted electrodes were carefully extracted and processed following Masson’s trichrome and H&E staining protocols (figure 7). Representative images demonstrated no observable nerve damage induced by electrodes (figures 7(A) and (B), top left). Masson’s trichrome staining revealed collagen encapsulation surrounding the entire microwire structure, with observable tissue ingrowth around individual strands of microwire bundles (figure 7(A), bottom left). H&E staining revealed a chronic immune response to the implanted gold microwires (figure 7(B), bottom left). Inflammatory cells were observed to surround the microwire bundles and infiltrate around the borders of the individual microwires, suggesting a persistent inflammatory response up to 12 weeks after electrode implantation. Overall, the observed foreign body response, characterized by collagen encapsulation and chronic inflammatory cell presence, aligns with expected tissue reactions to implanted materials and falls within typical biocompatibility parameters (Anderson *et al* 2008). Additional studies will be required in the future establish conclusive biocompatibility.

## 4. Discussion

Implanted Au-HMSEs were successful in electrically stimulating nerves for motor activation and recording evoked muscle activity (figure 3). Intramuscular EMG recordings were used to evaluate the evoked muscle activity in response to sciatic nerve stimulation and demonstrated a strong relationship with input stimulation amplitude (figure 3(B)). Furthermore, ankle torque measurements were recorded to complement EMG recordings in representing the overall motor output in response to electrical stimulation of the sciatic nerve (figure 3(C)). Evoked motor responses corresponding to intramuscular EMG and peak torque measurements correlated with the input stimulation. However, the signal strength and consistency in both measurements for motor responses varied substantially throughout the 8 weeks study for all stimulation configurations (figures 4 and 5(B)). In some instances, large motor responses that were captured in gastrocnemius EMG recordings during plantar flexion may originate from other non-selectively activated muscles. During plantar flexion, minimal activity in EMG recordings may be due to the recruitment of other muscles in response to nerve stimulation, apart from the gastrocnemius muscle, or the co-contraction of multiple activated muscles (Grill and Mortimer 1996, 2000).

Electrode migration is a primary concern when evaluating a neuromodulation treatment. Lead migration is multi-faceted, including changes in posture, and can occur immediately following surgical implantation (Osborne *et al* 2011, Kim 2013, Eldabe *et al* 2016). An open-helix electrode design was selected to provide continuous longitudinal strain relief and higher surface area to encourage tissue encapsulation and promote tissue ingrowth for further stabilize the electrode within the implant site (Howe *et al* 2024). The final assembly (3 mm in length, 0.6 mm in diameter) was formed by coiling 25 *µ*m gold bonding microwires. Similar to the principle involved in cables and ropes, the additional benefit of coiling wires, compared to a straight wire of similar dimensions, includes improvements to mechanical properties, such as flexibility and fatigue resistance (Cardou and Jolicoeur 1997, Costello 1997, Xiang *et al* 2015, Toossi *et al* 2017). Coiled wires are commonly used in the auxiliary portions of percutaneous leads (portion not in contact with the target tissue) to provide superb strain relief. The helical designs have also been adapted to improve tissue anchoring and reduce migration for functional electrical stimulation systems (Bowman and Erickson 1985, Handa *et al* 1989, Scheiner *et al* 1994). In these studies, implanted electrodes were used to evoke motor responses electrically and have demonstrated reliable performance months after implantation (Handa *et al* 1989, Kagaya *et al* 1998, Knutson *et al* 2002). In theory, these helical designs were developed to increase the available surface area for improved fibrotic tissue encapsulation of the electrode to stabilize implants and reduce lead migration (Peterson *et al* 1994, Kapural *et al* 2018). Although early designs aimed to improve device anchoring into muscle tissue, they may serve as a foundation for advancing novel designs in neural interfacing (Rauck *et al* 2014, Ilfeld *et al* 2017a). Coiled designs have been successfully used for percutaneous implantation in therapeutic neuromodulation applications as well (Chae *et al* 2013, Wilson *et al* 2014, Ilfeld *et al* 2017b, Gabriel *et al* 2019). However, in our study, the 3 mm helical design did not fully prevent *in vivo* electrode migration. This suggests that while the open-helix concept can encourage tissue integration, the implemented length may have been insufficient to realize the complete anchoring benefits described in previous literature. Additional testing will be required to fully assess this the proposed benefits of the open-helix design.

The Au-HMSE platform was initially designed for percutaneous implantation using syringe-needle guidance (Trevathan *et al* 2019, Dalrymple *et al* 2021). However, a more invasive approach was necessary to measure the long-term *in vivo* performance, including the electrode-tissue impedance, requiring an open-cut-down procedure to implant the head cap and leads (Atkinson *et al* 2022). This model allowed for both sending electrical signals to the implanted electrodes for stimulation and receiving signals from the electrodes for measuring electrode-tissue impedances and EMG activity (figure 1). Continuous *in-vivo* electrode-tissue impedance monitoring provides insights into the electrochemical properties at the electrode-tissue interface. A lower electrode-tissue impedance may be preferred for neuromodulation applications and has been used to indicate hardware functionality during treatment (Kumar *et al* 2006, Mullins *et al* 2023). However, extremely low impedance values may also suggest electrode shorting or loss of insulation, which can hamper the device’s performance and function (Lenis *et al* 2013, Swerdlow *et al* 2020, Shepherd *et al* 2021). Throughout 8 weeks, the average *in vivo* electrode-tissue impedances for all implanted electrodes were reported at 2.65 ± 0.15 kΩ (figure 2). This impedance level is consistent with previously reported values for chronic stimulation electrodes, supporting the suitability of the Au-HMSE design for stable long-term use (Grill and Mortimer 1994, Payne *et al* 2020, Mughrabi *et al* 2021).

Electrodes with an electrode-tissue impedance >10 kΩ were found to be unsuccessful in eliciting an evoked motor response when stimulating the sciatic nerve in a monopolar configuration. This elevated impedance may be due to several factors not extensively evaluated in this study, including lead migration, damage to the lead/electrode, or tissue remodeling, such as fibrosis (Alò *et al* 2006, Grill and Mortimer 1994, Kumar *et al* 2006). With this hypothesis in mind, the relationship between ‘viable’ electrodes implanted on the sciatic nerve with an electrode-tissue impedance <10 kΩ (7/8 by the end of the study) and its respective motor thresholds was evaluated. However, a weak correlation was found between these variables (*r* = 0.17; *p* < 0.08). Although the electrode-tissue impedance may be an accessible metric that informs the neural interface’s physical relation to its surrounding tissue, it fails to provide functional information regarding activation of nerve tissue (Shepherd *et al* 2021).

Despite achieving a large surface area, there were immediate trade-offs for effective electrical stimulation of the sciatic nerve. The implanted electrodes were not insulated and had a large surface area in contact with the sciatic nerve, approximately 0.6 mm^2^. This contact area may be larger if leads were repositioned around the nerve as a result of migration, leading to multiple nerve segments in contact with the electrode and susceptible to electrical stimulation. The inconsistencies observed in motor activation may be caused by electrical stimulation of different branches of the sciatic nerve. These branches may respond to the stimulation at varying intensities and could originate from either the main sciatic nerve or the individual nerve segments at the trifurcation (Grill and Mortimer 1996, Castro *et al* 2008). For future preclinical work and translation to clinical neuromodulation, careful electrode placement or additional insulation might be needed to avoid unwanted activation of adjacent tissues.

Interestingly, motor thresholds used to evoke an ankle response remained consistent, with minimal changes throughout 8 weeks for all stimulation amplitudes (figures 6(A) and (B)). However, the success rates in evoking an ankle response were not as consistent and varied throughout the study (figure 6(C)). At week 8, all stimulation configurations achieved >62% success rate in eliciting a motor response. Increasing the stimulation amplitudes over the 2.5 mA limit imposed in this study may lead to a response for cases where an evoked motor response was not achieved. This limitation was initially set from prior pilot studies (data not shown) to avoid potential tissue damage associated with high-charge injection (Cogan *et al* 2016).

Several precautionary steps limited optimal evaluation of the *in vivo* chronic performance of the Au-HMSEs, including a skull-mounted head cap (possibility of dislodgement), the use of soldered joints (wires may detach from connector pins), and mechanically secured interconnects used to attach two different wire materials. Furthermore, soldered leads at the connector pin may break within the cement-silicone package, leading to signal loss for that respective wire or potential shorting to other leads. Skull-mounted head caps may fail due to uncontrolled animal behavior that leads to physical dislodgement (subjects, *n* = 2, were removed and not reported in this study as a result of head cap failures throughout this study). Abnormal electrode-tissue impedances (>50 kΩ) suggested mechanical issues such as interconnect failure, wire breakage, or connector issues. These failure modes are common in pre-clinical neural interface studies and are difficult to overcome, especially given additional challenges unique to small animal experimentation. These results suggest the need for more robust interconnect solutions for chronically implanted leads in rodents or reducing the need for them altogether by moving to untethered approaches, such as wireless or transcutaneous interfacing (Verma *et al* 2021, Won *et al* 2023). The variability in stimulation success observed here suggests that, without further design optimizations, the HMSE could yield unpredictable outcomes in future preclinical and clinical studies. Ensuring consistent nerve contact and minimizing electrode migration will be critical to avoid the need for adjustments and electrode re-implantation.

Future work should incorporate other metals more commonly used in neuromodulation electrodes, such as platinum-iridium, as gold has been shown to have a lower electrochemical performance while being more susceptible to corrosion (White and Gross 1974, Cherevko *et al* 2014, Woodward 2014, Jovanovič *et al* 2018, Puglia and Bowen 2022). Unfortunately, intraoperative baseline data collection was not obtained as this invasive approach required lidocaine application at incision sites, as recommended by a veterinarian consult. The ability to elicit motor activity was obstructed during recovery following surgery. Thus, initial measurements were obtained at 1 week after implantation.

Future studies should be performed to evaluate the chronic immune response to implanted helical microwires. Although fibrotic encapsulation and tissue ingrowth was observed throughout the open-helix structure, infiltration of inflammatory cells were apparent within the center of the helix of the implanted electrodes (figure 7). Overall, our histological findings were ‘unremarkable’ in that they demonstrated an expected and typical foreign body response to an implanted device (Anderson *et al* 2008). No notable gross tissue abnormalities were observed at the implant sites prior to histology, aside from the expected fibrous encapsulation common to all implants.

Finally, while prior studies have shown that helical structures can help limit migration (Daly *et al* 2001, Ilfeld *et al* 2017a), we observed some migration and variable efficacy. In a clinical setting (e.g. VNS or chronic pain neuromodulation), such variability could result in inconsistent therapeutic outcomes and might require frequent recalibration or even re-implantation of electrodes if performance degrades. This may be due to the limited helical length of our devices, which likely did not provide adequate anchoring or strain relief, paired with their use in the periphery where limb movement can induce migration and lead breakage (Phillips *et al* 2004, Pena *et al* 2017). Future designs could incorporate longer coiling structures, varied helical coil diameters, additional tension loops, and sutures, as commonly used in peripheral nerve electrode implants, to enhance stability.

## 5. Conclusion

This study reports the reliability and functional performance of implanted Au-HMSEs for long-term sciatic nerve stimulation. The Au-HMSE architecture was designed to maximize the geometric surface area through a simplified electrode manufacturing procedure. This study aimed to evaluate its potential as a neural interface for chronic applications. Implanted Au-HMSEs had stable *in vivo* electrode-tissue impedances and continued to elicit stimulation-evoked motor responses up to 8 weeks after initial implantation. The histological evaluation was ‘unremarkable’ and demonstrated an expected fibrotic tissue encapsulation and tissue infiltration throughout Au-HMSEs 12 weeks after implantation. Future studies will focus on developing and assessing minimally invasive procedures, such as transcutaneous applications, to reduce percutaneous lead exposures while maintaining effective nerve stimulation. In conclusion, this study provides important insights into the functionality and stability of a neural interface consisting of Au-HMSEs, providing additional tools for researchers and medical professionals to evaluate state-of-the-art neuromodulation therapies, such as managing refractory chronic pain.

## Data Availability

All data that support the findings of this study are included within the article (and any supplementary files).
